# How does ethnic minority youth's dual self‐identification affect the formation of interethnic ties in friendship networks?

**DOI:** 10.1111/jora.70168

**Published:** 2026-03-11

**Authors:** Lexin Chen, Tobias H. Stark, Tom Nijs, Eva Jaspers

**Affiliations:** ^1^ Universiteit Utrecht European Research Centre on Migration and Ethnic Relations Utrecht The Netherlands; ^2^ Erasmus University Rotterdam Erasmus School of Social and Behavioral Sciences Utrecht The Netherlands; ^3^ Universiteit Utrecht Afdeling Sociologie Utrecht The Netherlands

**Keywords:** dual identifiers, ethnic identification, friendship, interethnic relations, schools, social networks

## Abstract

In an increasingly ethnically diverse Europe, this study examined the potential of dual identifiers, those identifying with both a national majority and an ethnic minority, such as German–Turkish individuals, to facilitate integration. As members of two groups, dual identifiers may be in the advantageous position to form more interethnic connections in ethnically diverse social networks. We propose that dual identifiers' intergroup behavior and their attractiveness as friends depends on how they construct their dual identity, such as identifying with both identities equally (compartmentalization), identifying more with the majority group (dominance‐majority), or more with the minority group (dominance‐minority). We analyzed three waves of German school data (averagely 1965 students per wave, 45% dual identifiers). Longitudinal social network analysis (stochastic actor–oriented models) indicated that dual identifiers primarily befriended peers from their mono‐minority group rather than forming connections to both groups they belong to. Analyses that took the different constructions of dual identity into account further showed that (1) stronger national identification did not alter friendship preferences but increased acceptance by the majority group; (2) mono‐majority identifiers treated dual and mono‐minority identifiers similarly; and (3) different types of dual identifiers exhibited similar friendship patterns, suggesting that identity construction did not significantly correlate with network preferences. These findings challenge assumptions that dual identifiers can connect different ethnic groups in interethnic networks, highlighting the complexity of interethnic social ties.

## INTRODUCTION

Research has shown that the majority of second‐ and third‐generation migrant youth in Europe express a sense of belonging to both the ethnic group of their (grand)parents and their country of residence (Fleischmann & Phalet, 2018). For example, in Germany, the descendants of Turkish immigrants may feel they belong to both the German national group and the Turkish ethnic group, thereby identifying themselves as national‐ethnic majority‐minority dual identifiers (hereafter referred to as “dual identifiers”). Despite representing a substantial group, there is limited research on their potential role in promoting ethnic integration—a critical issue in many European countries, where increasingly ethnically, culturally, and religiously diverse populations are faced with heightened tensions between different ethnic groups (Koopmans, 2015). As members of both the national majority and an ethnic minority group, it has been hypothesized that dual identifiers have the potential to form connections with multiple ethnic groups, thereby promoting ethnic integration and improving intergroup relations (Love & Levy, 2019).

There remains a scientific gap in understanding how the varying cognitive constructions of dual identities of adolescents with a migration background impact both their own social behavior and the behavior of others toward them (Verkuyten, 2018). While previous studies have predominantly explored the cognitive construction of dual identities (i.e., how people represent their multiple category identities, such as some individuals with stronger national identification than ethnic identification; see Brewer, 1991; Roccas & Brewer, 2002; Tajfel & Turner, 1979), and the (mis)perceptions of dual identifiers in terms of belonging, authenticity, and loyalty (e.g., Benet‐Martínez et al., 2002; Benet‐Martínez & Haritatos, 2005; Cheng et al., 2006; Fuligni et al., 2005; Nguyen & Benet‐Martínez, 2013; Phinney, 2013; Verkuyten, 2005), little research has examined how the cognitive construction of dual identities specifically shapes social network integration and peer friendship formation in adolescence.

Research has shown that intergroup attitudes follow distinct developmental trajectories, with adolescence being a key period where these attitudes shift due to both individual experiences and social influences (Van Zalk & Kerr, 2014). At the same time, research has shown that dual identification can influence prejudice levels (Levy et al., 2019; Levy, Saguy, et al., 2017). When group boundaries are perceived as permeable, dual identity can reduce prejudice and likely also group boundaries in social behaviors; however, when boundaries are seen as impermeable, dual identity may not have the same effect (González & Brown, 2006). Thus, the potential of dual identifiers to form intergroup ties may vary depending on how they construct and represent their dual identities. For example, those who identify more strongly with the national majority group than the ethnic minority group may be preferred as friends by the majority group members (e.g., Jugert et al., 2018; Leszczensky et al., 2016). At the same time, they might be less likely to form ties with peers of their ethnic minority group due to their relatively weak ethnic identification. We aim to contribute to this literature by examining how dual identifiers' various ways of self‐identification are linked to both their own preferences and others' preferences for friendship choices within interethnic social networks during adolescence.

We explored the role of dual identifiers in fostering intergroup relations within interethnic school friendship networks, specifically examining whether dual identifiers were more likely to form ties to multiple ethnic groups, and whether and how different construction of dual identities was linked to the friendship preferences of both dual and mono‐identifiers. The present analysis utilized the German Friendship and Identity School (FIS) dataset, a comprehensive longitudinal dataset encompassing three waves of individual‐ and relation‐level responses from approximately 2000 students across nine secondary schools in Germany (Leszczensky et al., 2022). The school context was examined for three primary reasons: (1) schools serve as pivotal environments for the social construction of national and ethnic identification among students (Banks & Banks, 2019; Huber & Kitson, 2020; Verkuyten & Thijs, 2002); (2) educational institutions provide a mandatory setting in which students from diverse ethnic backgrounds engage in communication, socialization, and cultural exchange, fostering the development of social connections across ethnic groups (e.g., Boda, 2019; Boda et al., 2020; Boda & Néray, 2015; Leszczensky et al., 2016; Leszczensky & Pink, 2019); and (3) compared to childhood or adulthood, peer norms have the strongest effect on social behaviors during adolescence (Brechwald & Prinstein, 2011). If interethnic friendships become common in peer networks, they can set new social norms that have lasting impact beyond the school context and age.

### National‐ethnic majority‐minority dual identifier

The term dual identifiers describes individuals who simultaneously maintain multiple identities, which may belong to the same or different dimensions (e.g., Dovidio et al., 2007; Fleischmann & Phalet, 2016; Roccas & Brewer, 2002). This study focuses specifically on dual identifiers within the same dimension, namely the national‐ethnic dimension (Jugert et al., 2022). We use “ethnic” as referring to the shared belief in a common lineage and ancestry (Weber, 1968). In our case, native Germans are thus considered ethnic majority members due to their majority ancestry. In contrast, for descendants of immigrants, *ethnic identification* reflects their commitment to their family's origin or cultural heritage, whereas *national identification* pertains to their relationship with the country of residence or the majority group (Fleischmann & Phalet, 2016; Verkuyten & Martinovic, 2012).

Our conceptualization of dual identity is defined based on the subjective self‐identification of individuals, instead of an objective criteria such as family origin, a method that has been criticized for taking an essentialist approach (see Haslam et al., 2000; Keller, 2005; Verkuyten, 2003). Objective categorization overlooks subjective experiences and feelings, especially for dual identifiers who do not have a national majority ancestry. For example, children of Turkish immigrants who are born and raised in Germany often self‐identify as both German and Turkish, without having ethnic German ancestry. While maintaining strong and stable connections with the minority culture of their family, they can participate in the German society without the language or cultural barrier their parents faced; however, this participation in the German society often occurs in the context of persistent structural inequalities and exclusionary dynamics (e.g. Barwick & Beaman, 2019; Demmrich & Akgül, 2020; Fernandez‐Kelly, 2012). This dual engagement shapes their social identity and behaviors (e.g., Cárdenas et al., 2021; Levy et al., 2023; Tseung‐Wong et al., 2019). However, existing network studies typically categorized dual identifiers as minority members based on their family origin (e.g., Leszczensky, 2013; Leszczensky et al., 2016; Leszczensky & Pink, 2019; Stark & Flache, 2012). Even social network studies that considered participants' self‐identification categorized descendants of immigrants either as majority or minority members, and did not allow for dual identification (Stark, 2015; Zingora et al., 2020). Although prior research has begun to examine the role of dual identifiers in interethnic social networks (Jugert et al., 2018), key questions remain regarding whether and how different constructions of dual identity influence homophily based friendship formation in interethnic peer networks.

### Friendship preferences of and for dual identifiers

Dual identifiers may be in an advantageous position to more easily form connections to multiple ethnic groups (Love & Levy, 2019). Homophily, the tendency of individuals to associate and bond with similar others, is a well‐established mechanism driving the formation of friendship ties in social networks (e.g., Goodreau et al., 2009; Leszczensky, 2013; Leszczensky et al., 2016; Levy et al., 2019; McPherson et al., 2001). This homophily mechanism may allow dual identifiers to establish and maintain social network ties with both the national majority group and members of their ethnic minority group because they can be seen as members of both groups. For example, both German mono‐identifiers and Turkish mono‐identifiers might regard German‐Turkish dual identifiers as in‐group members, increasing their likelihood to form social ties with them.

It is important to note that our focus is not on whether dual identifiers create bridges between segregated groups, but rather on whether they are more likely than mono‐identifiers to form ties to multiple groups. Given the dynamic nature of social networks, it is possible that a majority group friend and a minority group friend of a dual identifier are also friends with each other in our data. In such cases, the dual identifier may not represent a bridge in the technical sense (between unconnected actors). However, given the well‐established tendency for triadic closure (a friend of a friend is a friend), it is plausible that the majority and minority group students were first only friends with the dual identifier and later became friends with each other. Focusing solely on the potential of dual identifiers to create ties to unconnected people in a network would underestimate their potential to form ties with members of different groups.

Although dual identifiers have not been explicitly examined in most previous social network research on interethnic friendship formation (for an exception, see Jugert et al., 2018), there is some evidence that points in the direction of their potential to form ties to multiple ethnic groups. Leszczensky (2013) found that students with a migration background with stronger national identification were more strongly preferred as friends by majority students. Similarly, Leszczensky et al. (2016) demonstrated that descendants of immigrants with higher national identification were preferred more by majority students, and that their national identification did not impact their friendship relations with other students with a migration background. These findings highlight the potential of dual identifiers to connect to the national majority and ethnic minority groups, as they are more likely to be perceived as in‐group members by majority students due to their strong national identification while they remain connected with other‐minority students. Jugert et al. (2018) concluded that descendants of immigrants who had solely national identification or dual identification were preferred as friends by both ethnic majority and minority students. Dual identifiers, in contrast, befriended ethnic majority students and other dual identifiers but were less likely to befriend ethnic minority identifiers. However, as pointed out by the authors (Jugert et al., 2018, p. 390), this study did not account for ethnic homophily in students' friendship choices. Instead of testing whether dual identifiers would form friendships with mono‐minority identifiers of their own ethnic group, the study tested their tendency to form friendships with mono‐minority identifiers from all minority groups. In conclusion, prior studies suggest that dual identifiers have the potential to connect to differing identities, but it remains particularly unclear if dual identifiers form connections to minority members of their own ethnicity. Accordingly, we expect:

Hypothesis 1a Mono‐majority identifiers have a stronger preference for friendships with dual identifiers than for friendships with mono‐minority identifiers.

Hypothesis 1b Mono‐minority identifiers have a stronger preference for friendships with dual identifiers of the same ethnicity than for friendships with mono‐majority identifiers.

In addition, we expect that dual identifiers exhibit stronger preferences for establishing friendship ties with individuals from the two groups they identify with than with peers who belong to other groups.

Hypothesis 1c Dual identifiers have a stronger preference for friendships with mono‐minority identifiers of the same ethnicity, dual identifiers of the same ethnicity, and mono‐majority identifiers than for friendships with members of other ethnicities.

### How construction of dual identities is associated with friendship preferences

Dual identifiers may adopt various strategies for organizing their dual identities (see Amiot et al., 2007; Benet‐Martínez & Haritatos, 2005; Bhabha, 2012; Brewer, 1991; Hong et al., 2000; Roccas & Brewer, 2002), leading to potentially different implications for the homophily mechanism. The construction of dual identities may affect friendship preferences and certain types of dual identities may attract friendships from mono‐identifiers differently.

According to the theoretical framework of Roccas and Brewer (2002), dual identifiers can be categorized into four types that differ in how the two identities are cognitively organized. While the social identity complexity model was developed in adult populations, adolescents in early to mid‐adolescence are developmentally capable of reflecting on and integrating multiple social identities, particularly in peer‐related contexts (Quintana, 1994; Umaña‐Taylor et al., 2014). Given the centrality of both identity exploration and peer relationships during this period, applying this framework to adolescent dual identifiers is appropriate and theoretically consistent with the developmental tasks of adolescence. A summary of the definitions and expectations of the four types of dual identifiers is given in Table 1.

**TABLE 1:** Dual identity organization according to the social identity complexity model, using German–Turkish dual identifiers as an example.

Conceptualization	Interpretation	Hypothesized friendship preferences
Intersection	“I self‐identify as being German‐Turkish, but not or weakly as being German or Turkish”	All German–Turkish dual identifiers
Dominance‐majority	“I feel more strongly as being German and less so as being German‐Turkish or Turkish”	Mono‐German identifiers, all types of German–Turkish dual identifiers
Dominance‐minority	“I feel more strongly as being Turkish and less so as being German‐Turkish or German”	Mono‐Turkish identifiers, and all types of German–Turkish dual identifiers
Compartmentalization	“I self‐identify as being German and Turkish equally, but not or weakly as being German‐Turkish”	Mono‐German identifiers, Mono‐Turkish identifiers, and all types of German–Turkish dual identifiers

Dual identifiers with intersecting identities consider themselves as belonging to a compound category, forming “a single, unique social identity with properties that make it distinct from either of the larger categories from which it is derived” (Roccas & Brewer, 2002, p. 90).

Dominance is another way dual identifiers may organize their dual identities. This type designates one identity as primary, while the other is subordinated to it, establishing a hierarchical structure of identities (Roccas & Brewer, 2002). Dual identifiers of this type typically consider both dual identifiers and mono‐identifiers of the primary group as in‐group members. Considering the majority/minority division, there are two types of dual identifiers under this category: dominance‐majority and dominance‐minority dual identifiers. For example, a dominance‐majority German–Turkish dual identifier, who identifies more strongly as German than as Turkish, would consider mono‐identifying Germans and all types of German–Turkish dual identifiers as in‐group members. In contrast, dominance‐minority dual identifiers would have a preference for all types of same‐ethnic dual identifiers and same‐ethnic mono‐minority identifiers.

We combine merger and compartmentalization of the Roccas and Brewer (2002) typology as the third way in which dual identifiers may organize their dual identities. These represent stable (merger) or context‐dependent (compartmentalization) versions of the most inclusive form of dual identification, where “ingroup identification is extended to others who share any of one's important social category memberships” (Roccas & Brewer, 2002). Thus, for a German‐Turkish individual, in‐group members would include all types of German‐Turkish individuals, mono‐Germans, and mono‐Turkish individuals. Dual identifiers of these types do not prioritize any identity like the dominance dual identifiers, and they regard themselves as members of both groups unlike the intersection dual identifiers. We choose the term “compartmentalization” to describe dual identifiers who consider individuals identifying with any of the groups they belong to as in‐group members. To differentiate between intersection and compartmentalization dual identifiers, we propose that intersection dual identifiers weakly identify as members of both groups but strongly identify as the members of an intersectional group, or a special compound. In contrast, compartmentalization dual identifiers would strongly identify as members of both groups but weakly identify as the member of the intersectional group.

In this study, we focus our theoretical development and hypotheses on dominance‐minority and compartmentalized dual identifiers. While the full typology informs our conceptual approach, we center our analysis on these two types due to data limitations. Although we had data on nearly 2000 students, only very small numbers of students fell into the intersection and dominance‐majority categories. Accordingly, we expect:

Hypothesis 2 Dominance‐minority dual identifiers have a stronger preference for friendships with mono‐minority identifiers of the same ethnicity and dual identifiers of the same ethnicity than for mono‐majority identifiers.

Hypothesis 3 Compartmentalization dual identifiers have a stronger friendship preference for mono‐majority identifiers, dual identifiers of the same ethnicity, and mono‐minority identifiers of the same ethnicity than other identifiers.

### Mono‐identifiers' preference for different types of dual identifiers

To successfully create connections with different ethnic groups, dual identifiers must also be chosen as friends by mono‐majority identifiers and mono‐minority identifiers. The homophily mechanism applies to mono‐identifiers straightforwardly in that they prefer other mono‐identifiers of the same group as friends (McPherson et al., 2001; Moody, 2001). For the mono‐majority group, next to a preference for other mono‐majority identifiers, several studies (Jugert et al., 2018; Leszczensky, 2013; Leszczensky et al., 2016) revealed that mono‐majority students also befriend students with a migration background when they identify strongly with the national group. This suggests that dual identifiers who have relatively strong national identification, that is, compartmentalization dual identifiers, may also be considered as in‐group members by mono‐majority identifiers. Accordingly, we expect:

Hypothesis 4 Mono‐majority identifiers have a stronger preference for friendships with mono‐majority identifiers, and compartmentalization dual identifiers than with other identifiers.

In parallel, mono‐minority identifiers should prefer mono‐minority identifiers of the same ethnicity as in‐group members according to the homophily mechanism. In addition, they may also prefer dual identifiers of the same ethnicity who have relatively strong ethnic ingroup identification, that is, dominance‐minority and compartmentalization dual identifiers.

Hypothesis 5 Mono‐minority identifiers have a stronger preference for friendships with same‐ethnic mono‐minority identifiers, dominance‐minority dual identifiers, and compartmentalization dual identifiers than for friendships with other identifiers.

## DATA AND METHODS

### Data

We utilized the German Friendship and Identity School data (FIS), a longitudinal dataset spanning six waves and collected over four and a half years across 10 secondary schools in Germany (Leszczensky et al., 2022). Due to non‐participation of certain grades in the survey from the fourth wave onward, we restricted our analysis to data from the first three waves. There were 29 grades in total, and each grade contained 2 to 5 classes. See descriptive statistics of this dataset in Table 2.

**TABLE 2:** Descriptive statistics of FIS data.

	Wave 1	Wave 2	Wave 3
Number of students	1799	2024	2071
Average number of students per grade	62	70	71
Gender composition: proportion female	52.2%	53.3%	52.7%
Majority group: proportion German	42.9%	40.3%	39.1%
Ethnic composition			
Germany	772	816	810
Turkey	458	504	498
Former Soviet Union	100	115	119
Poland	97	117	117
Former Yugoslavia	40	55	56
Italy	49	77	71
Lebanon	28	25	24
Greece	26	28	33
North Africa	32	38	31
South Africa	20	18	20
Latin America and the Caribbean	6	5	11
North America and Oceania	5	11	7
Southern Asia	27	33	38
Western Asia	16	15	24
Other Asia	11	15	17
Other Eastern Europe	19	26	24
Other Southern Europe	45	65	63
Other Europe	38	45	50

#### Dependent variable

To measure the friendship network, participants could nominate up to 10 best friends within their grade. Students were permitted to nominate friends outside their classes but not outside their grades. Accordingly, we modeled the grade‐level friendship networks as the dependent variable. The majority of students nominated between 4 and 8 friends. The mean friendship nominations across the three waves were 5.72, 5.53, and 5.23 respectively, indicating a decreasing trend wherein students tend to name fewer friends as they progress through school.

#### Independent variable

Students' self‐identification was operationalized through responses to survey questions measuring both national and ethnic identification. Each construct was assessed using six items adapted from Leszczensk and Santiago (2015). For national identification, participants responded to what extent the statements such as “I am satisfied with being part of Germany” or “I am happy to belong to Germany” apply to them. Ethnic identification was measured using parallel items reflecting the participant's family's country of origin (Cronbach's alpha is larger than 0.75 for all groups, see Leszczensky et al., 2022; see Online Supplement [Appendix 1] for all items). Responses for all items were recorded on a five‐point Likert scale. Responses for each scale were averaged with higher values indicating stronger national or ethnic identification.

We further leveraged the strengths of national and ethnic identification to classify participants into distinct types of identifiers (Schlette et al., 2024), following the theoretical framework established by Roccas and Brewer (2002). Table 3 provides a detailed classification scheme for categorizing participants into these identifier types. Participants with a strong identification with one group and a weak association with the other (i.e., one rated between 3 and 5, the other between 1 and 2) were categorized as either mono‐majority identifiers or mono‐minority identifiers, depending on which identification was stronger. Participants who strongly and equally identify with both groups (both rated between 3 and 5, and the difference is less than 1) were classified as compartmentalization dual identifiers. Those with a strong identification with one group and a moderately weak association with the other (the stronger rated between 3 and 5, the weaker larger than 2, and the difference is equal or larger than 1) were classified as either dominance‐majority dual identifiers or dominance‐minority dual identifiers, based on which identification was predominant. Participants who moderately weakly identified with both groups (both rated between 2 and 3) were classified as intersection dual identifiers. To distinguish intersection dual identifiers from non‐identifiers (those with weak identification with both groups), an additional survey question was used: “*Some people see themselves as German, others as Turkish, for example, and still others as German‐Turkish. How is it with you? How do you see yourself*?” Respondents were able to provide an open answers or select from predefined options (German, Turkish, German‐Turkish, Kurdish, German‐Kurdish, Italian, German‐Italian, Polish, German‐Polish). Only participants selecting hybrid responses (e.g., German‐Turkish) or providing hybrid open answers were categorized as intersection dual identifiers. Non‐identifiers were added as a control variable to our analyses.

**TABLE 3:** Classification table of dual identifiers.

Strength of ethnic identification	Strength of national identification			
	1 −< 2	2 −< 3	3 −< 4	4 −< 5
1 −< 2	Non‐identifiers	Non‐identifiers	Mono‐majority	Mono‐majority
2 −< 3	Non‐identifiers	Intersection^a^	Dominance‐majority	Dominance‐majority
3 −< 4	Mono‐minority	Dominance‐minority	Compartmentalization	Dominance‐majority
4 −< 5	Mono‐minority	Dominance‐minority	Dominance‐minority	Compartmentalization

^a^ An additional question is used to classify participants with this response pattern into the intersection type.

To justify our identity classification, we conducted two robustness checks, and the results are shown in Online Supplement [Appendix 2 (Table A1, Table A2)]. In the main analysis, students were categorized as dominance‐majority or dominance‐minority dual identifiers only when the difference between their national and ethnic identification strengths exceeded 1, reflecting a substantial imbalance indicative of one identity meaningfully dominating the other. In the first robustness check, we relaxed this condition and classified students as dominance‐type whenever one identity was stronger than the other, regardless of the magnitude of the difference. The results remained largely consistent with those from the main analysis, suggesting that the substantive conclusions do not hinge on this specific threshold. In the second robustness check, we slightly lowered the cutoff point for distinguishing mono‐identifiers from dual identifiers (from 2.0 to 1.8), which reclassified a small number of students. As some dual identifier subgroups were not included in the model, this adjustment led to more missing cases and a slight reduction in statistical power. While some previously significant effects became non‐significant (though p‐values remained close to conventional thresholds), the overall pattern of results remained similar. These checks together support the robustness and theoretical meaningfulness of our original operationalization.

To determine the ethnic group membership of dual identifiers and minority students, we used answers about the country of birth of participants' parents or grandparents. Participants who reported that both they and their parents or grandparents were born in Germany were categorized as the majority group. If the participants reported another country of birth for themselves or their (grand)parents, they were assigned to this ethnicity. If different birth countries were reported, we assigned them to the one they chose to be more important.

Descriptive data for three waves are presented in Table 4. Across all three waves, dual identifiers comprise approximately 40% of the total population. However, the numbers of intersection and dominance‐majority identifiers are very small in all three waves, with 17 grades lacking intersection dual identifiers and 4 grades lacking dominance‐majority dual identifiers.

**TABLE 4:** Number of students by categorization per wave and average strength of national/ethnic identification per category.

	Wave 1	Wave 2	Wave 3	Average strength of national identification	Average strength of ethnic identification
Mono‐majority identifiers	819	894	895	3.57	3.73
Mono‐minority identifiers	105	155	173	1.44	4.65
Compartmentalization dual identifier	311	354	325	4.04	4.35
Intersection dual identifier	7	6	7	2.68	2.67
Dominance‐majority dual identifier	17	18	45	4.19	2.87
Dominance‐minority dual identifier	458	504	498	2.93	4.69
Non‐identifier	11	25	36	1.72	1.74

#### Control variable

Gender and a variable for the school class students belong to were included as control variables in the analysis due to the observed strong effects of gender homophily and class homophily in friendship networks (e.g. Boda, 2019; Jugert et al., 2018; Leszczensky et al., 2016; Leszczensky & Pink, 2019). Age or age homophily are not taken into consideration because the social networks are constructed at the grade level, meaning all students within each network are in the same school grade and thus of very similar age. In addition, we control for students' country of origin in the analysis, but the wide diversity and small sizes of many origin groups made it impractical to include separate ego and alter effects for each country without risking overfitting or convergence issues. Instead, we capture ethnic background through a same‐ethnicity effect and relevant interaction terms involving ethnic identity categories.

### Methodology

The R scripts necessary for replication and reproduction of the analyses are available here: (https://osf.io/5cbgv/?view_only=442601daf5334ace81f42f3e8999f075).

We employed stochastic actor–oriented models (SAOM) in the R‐package RSiena Version 1.4.13 (Ripley et al., 2024). SAOMs are an agent‐based analytical method designed to model dynamics in social networks observed over multiple instances (Snijders, 2005; Snijders et al., 2010; Veenstra et al., 2013), but it does not allow for definitive causal inference. These models simulate the evolution of networks by iteratively modeling actors' interdependent choices in forming and maintaining friendships. The conditional odds ratios for the friendship behavior of each type of ethnic self‐identification were calculated as the conditional log probabilities reflecting how individuals (egos) chose to establish friendship ties with others (alters) based on egos' and alters' ethnicity and their ethnic self‐identification. SAOMs are advantageous for testing our hypotheses as they allow for the control of well‐established relational effects (i.e., structural characteristics of the network itself) and proximity mechanisms (i.e., opportunities for interaction). Accounting for these mechanisms is vital to mitigate bias in estimating selection effects linked to individual preferences.

Because of significant heterogeneity within the dataset, we modeled each grade separately and conducted a random‐effects multivariate meta‐analysis to aggregate findings across multiple grades (e.g., Jugert et al., 2018). This facilitates the identification of consistent patterns and underlying sources of heterogeneity across different networks, thus contributing to a more nuanced and generalizable understanding of selection processes and other critical aspects of network evolution. Only those networks in which the analysis converged are included in the meta‐analysis. The SAOM's convergence process served as a natural filter, automatically removing networks in which the theoretically important tie patterns were insufficiently represented. Consequently, the grade networks included in the meta‐analysis are those where all identification groups and interaction patterns were present at levels that allowed stable estimation of the complex model terms. This procedure does not introduce bias; rather, it ensures that inferences are based only on networks capable of supporting the necessary relational configurations, thereby avoiding overstatement of results drawn from structurally limited data.

In the first SAOM, we treated dual identifiers as one group to test Hypotheses 1a, 1b, 1c. The model incorporated fundamental network structural effects to account for various relational mechanisms shaping adolescent networks, avoid biased estimates of ethnic homophily, and reduce potential confounding from unobserved actor‐level characteristics (Ripley et al., 2024; Leszczensky & Pink, 2019). We controlled for the outdegree effect, which captures students' general propensity to nominate classmates as friends, the reciprocity effect, which reflects the extent to which friendship nominations are mutual, the geometrically weighted edgewise shared partners (GWESP) effect, which accounts for students' tendency to befriend their friends' friends, and an interaction between reciprocity and GWESP effects, capturing variations in reciprocity within open and closed triads (Block, 2015). Finally, the outdegree‐activity, indegree‐popularity, and indegree‐activity effects represent key dynamics of network formation, acknowledging that some students are more sociable or popular than others.

In addition to these network structural effects, our model incorporated a set of covariates to account for various factors that may enhance the likelihood of friendship formation between students (Smith et al., 2014). These included ego and alter gender effects, the same‐gender effect, and being in the same‐class effect.

To test Hypothesis 1a, we compared mono‐majority identifiers' preference for dual identifiers with mono‐majority identifiers' preference for mono‐minority identifiers. Since mono‐majority identifiers were set as the reference category in our analysis, this group's preferences could be directly inferred from the alter effects of dual identifiers and mono‐minority identifiers. If the alter effect of dual identifiers was significantly larger than the alter effect of mono‐minority identifiers, Hypothesis 1a could be supported.

To test Hypothesis 1b, we compared the mono‐minority group's preference for dual identifiers of the same ethnicity to their preference for mono‐majority identifiers. To estimate the first, the three‐way interaction effect between ego (mono‐minority), alter (dual identifier), and same (ethnicity) was included in the model, along with the corresponding lower‐order two‐way interactions and main effects. This ensured that the model appropriately accounted for the individual and pairwise contributions of the interacting variables, allowing for a meaningful interpretation of the three‐way interaction. We calculated the mono‐minority group's preference for dual identifiers of the same ethnicity by the linear combination of the three‐way interaction effect and the lower‐order terms and compared this to the mono‐minority group's preference for mono‐majority identifiers. The latter was represented by the ego (mono‐minority) effect, given that mono‐majority identifiers were the reference category. If this difference was significantly positive, Hypothesis 1b could be supported.

Testing Hypothesis 1c required calculating dual identifiers' preferences for mono‐majority identifiers, same‐ethnic dual identifiers, and same‐ethnic mono‐minority identifiers separately. Dual identifiers' preference for the mono‐majority group was represented by ego (dual identifier) effect given that mono‐majority identifiers were the reference category. Dual identifiers' preference for same‐ethnic dual identifiers was represented by the three‐way interaction between ego (dual identifier), alter (dual identifier), and same (ethnicity), while their preference for same‐ethnic mono‐minority identifiers was embodied in the three‐way interaction between ego (dual identifier), alter (mono‐minority identifier), and same (ethnicity). If the three effects representing different preferences were all significantly positive, Hypothesis 1c could be supported.

In addition, we also included the three‐way interaction between ego (mono‐minority identifier), alter (mono‐minority identifier), and same (ethnicity), and the corresponding lower‐order terms because we assumed a strong same‐ethnic preference within mono‐minority groups.

To test varying preferences of different types of dual identifiers (Hypotheses 2, 3, 4, 5), we ran a second SAOM. This model included the same network structural effects and control covariates as the first model. Preliminary tests revealed that due to the extremely small sample sizes of intersection dual identifiers and dominance‐majority dual identifiers, it was not feasible to include these two groups in the analysis. Additionally, incorporating the ego and alter effects of non‐identifiers caused convergence issues, likely due to the small sample size of this group. Descriptive statistics indicated that many grades lacked intersection dual identifiers, dominance‐majority dual identifiers, and non‐identifiers. Excluding these groups did not introduce bias but rather improved model fit. We therefore excluded the ego and alter effects of these groups. By doing so, they were merged into the reference category. Full model specification and meta‐analysis results are shown in Online Supplement [Appendix 3 (Table A3)] in the online supplement.

To test Hypothesis 2 about dominance‐minority dual identifiers' preferences for same‐ethnic peers, we included three three‐way interaction effects (and the underlying two‐way interactions) between ego (dominance‐minority dual identifier), same (ethnicity), and alter being also dominance‐minority dual identifier, alter being compartmentalization dual identifier, or alter being mono‐minority identifier. Dominance‐minority dual identifiers' preferences for mono‐majority identifiers were represented by the ego (dominance‐minority dual identifier) effect because mono‐majority identifiers were the reference category.

To test Hypothesis 3, we needed to calculate compartmentalization dual identifiers' preferences for four groups. These were represented by three three‐way interactions between ego (compartmentalization dual identifier), same (ethnicity), and alter being also compartmentalization dual identifier, alter being dominance‐minority dual identifier, or alter being mono‐minority identifier. Compartmentalization dual identifiers' preferences for mono‐majority identifiers were represented by the ego (compartmentalization dual identifier) effect.

Testing Hypothesis 4 required estimating mono‐majority identifiers' preferences for all other groups. Because mono‐majority identifiers were the reference category, their preferences could be directly inferred from the alter effects for compartmentalization dual identifiers, dominance‐minority dual identifiers, and mono‐minority identifiers. As mono‐majority identifiers' preference for their own group served as the baseline, support for this hypothesis required a significantly positive alter effect for compartmentalization dual identifiers and significantly negative alter effects for dominance‐minority dual identifiers and mono‐minority identifiers.

Testing Hypothesis 5 required estimating mono‐minority identifiers' preferences for different types of identifiers from the same ethnic group. Therefore, we include three three‐way interactions between ego (mono‐minority identifiers), same (ethnicity), and alter being compartmentalization dual identifier, alter being dominance‐minority dual identifier, or alter being also mono‐minority identifier. If all three group preferences were significantly positive, Hypothesis 8 could be supported.

## RESULTS

### Descriptives

#### Descriptives of the 13 converged grade networks

We initially included 29 grades in the analysis, but only 13 grades were retained for the meta‐analysis because they successfully converged in the SAOM models. Non‐converging grades were not excluded arbitrarily but typically lacked sufficient ties between the identity types for estimating complex three‐way interactions. In such cases, the model produced irregular or inflated coefficients due to structural sparsity, making estimation unreliable. The 13 converged grade networks, by contrast, had adequate representation of relevant tie types and were thus suitable for robust estimation. We present wave‐level descriptive summaries in Table 5, averaged across the 13 grades, while detailed grade‐level tables by wave are provided in Online Supplement [Appendix 4 (Table A4, Table A5, Table A6)]. Notably, while the average number of mono‐minority to mono‐minority ties was low in Wave 1 (averagely only 1 per grade), it increased to 6 in Wave 2 and 8 in Wave 3, as shown in Table 5, providing sufficient information for estimating effects involving these groups.

**TABLE 5:** Wave‐level descriptives of the 13 grade networks included in the SAOM.

Variables	Wave		
	1	2	3
Average ethnic distribution (five largest groups)			
Germany	28	28	27
Turkey	20	22	23
Former Soviet Union	5	5	5
Poland	4	5	4
Italy	2	3	2
Average number of students	71	79	80
Average number of friends	497	489	474
Average number of ties from mono‐majority to mono‐majority	68	75	71
Average number of ties from mono‐majority to dual	56	57	50
Average number of ties from mono‐majority to mono‐minority	7	9	8
Average number of ties from dual to mono‐majority	58	55	43
Average number of ties from dual to dual	101	105	86
Average number of ties from dual to mono‐minority	10	21	20
Average number of ties from mono‐minority to mono‐majority	5	10	7
Average number of ties from mono‐minority to dual	11	20	21
Average number of ties from mono‐minority to mono‐minority	1	6	8

### Friendship preferences when dual identifiers are considered as one group

The first SAOM treated dual identifiers as one group, aiming to test Hypotheses 1a, 1b, 1c. SAOMs of 13 grades were retained for the meta‐analysis after excluding those that did not achieve acceptable convergence (around 0.25) or exhibited irregular coefficients (Ripley et al., 2024). The full SAOM output of the meta‐analysis is given in Table 6. The positive and significant effects of reciprocity and GWESP indicate that students tended to form mutual ties and closed triads, though the negative interaction between them suggests that reciprocal ties reduced the likelihood of triad closure. These are normal findings in friendship networks (e.g. Block, 2015; Preciado et al., 2012; Snijders & Baerveldt, 2003). Negative indegree‐popularity and indegree‐activity effects show that students with many incoming ties neither attracted nor initiated more ties. By contrast, the positive outdegree‐activity effect suggests that students who initiated many ties were likely to form even more. Strong gender and class homophily effects indicate a tendency to befriend similar peers, while the negative gender ego effect shows that female students were less likely to initiate ties than male students. A strong same‐ethnicity effect also points to pronounced ethnic homophily among mono‐majority students, the reference group.

**TABLE 6:** Meta‐analysis SAOM result: dual identifiers as a whole group.

Effects	Estimates	Standard error
outdegree (density)	−2.640***	0.075
reciprocity	1.680***	0.079
GWESP I −> K −> J (69)	1.776***	0.046
reciprocity × GWESP I −> K −> J (69)	−0.689***	0.070
indegree—popularity	−0.070***	0.007
outdegree—activity	0.017***	0.003
indegree—activity	−0.145***	0.014
m_minor alter	−0.034	0.058
m_minor ego	0.096	0.077
dual alter	0.018	0.020
dual ego	0.062	0.038
gender alter	0.009	0.011
gender ego	−0.022^†^	0.011
same gender	0.467***	0.033
same class	0.523***	0.026
same ethnicity	0.170***	0.037
m_minor ego × m_minor alter	0.101	0.153
dual ego × dual alter	0.001	0.030
m_minor ego × dual alter	−0.047	0.069
dual ego × m_minor alter	−0.037	0.074
m_minor ego × same ethnicity	0.139	0.098
m_minor alter × same ethnicity	0.083	0.099
dual ego × same ethnicity	−0.066^†^	0.034
dual alter × same ethnicity	0.038	0.045
m_minor ego × dual alter × same ethnicity	0.192^†^	0.110
m_minor ego × m_minor alter × same ethnicity	0.389	0.310
dual ego × dual alter × same ethnicity	0.097**	0.031
dual ego × m_minor alter × same ethnicity	0.231*	0.112

*Note*: GWESP: geometrically weighted edgewise shared partners; m_minor: mono‐minority identifiers; gender: 1 = female.

^†^ *p* < .1.

* *p* < .05.

** *p* < .01.

*** *p* < .001.

**FIGURE 1:**
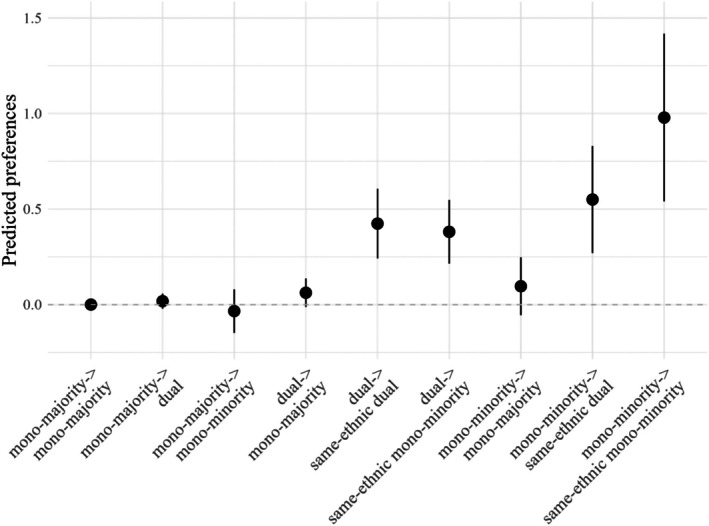
Group friendship preferences. Friendship preferences of different identifiers' groups are represented by point estimates along with 95% confidence intervals from meta‐analysis of 13 networks. The zero line represents the reference category, friendships between mono‐majority identifiers. Therefore, there is no confidence interval for mono‐majority identifiers' preference for mono‐majority identifiers. Differences between grade‐level networks and standard errors of estimates are reflected by the confidence intervals.

Figure 1 visualizes group preferences, which were calculated using linear combinations based on the model results. See Online Supplement [Appendix 5 (Table A7), Appendix 6 (Table A9)]. The “predicted preference” values reflect linear combinations of SAOM parameter estimates for different ego‐alter identity pairings, expressed on the log‐odds scale. These predictions are derived from the meta‐analytic model and reflect estimated tendencies toward tie formation, controlling for all other effects in the model. Each group's preference is represented by a point estimate and its confidence interval. If the lower limit is larger than 0, this preference was significantly positive. Mono‐majority identifiers formed the reference category which means their preferences for mono‐majority friends represents the baseline against which the other preferences are compared. A higher predicted value implies in practical terms, for example, a higher relative likelihood of a tie forming between a mono‐minority student and a dual identifier of the same ethnicity, compared to a baseline reference.

Hypothesis 1a proposed that mono‐majority identifiers prefer dual identifiers over mono‐minority identifiers. However, the first three estimates in Figure 1 demonstrate that mono‐majority identifiers did not differentiate between dual identifiers and mono‐minority identifiers, as the predicted values for both are non‐significantly different from the baseline. A direct z‐test (see Online Supplement [Appendix 6 (Table A10)]) showed that the two predicted values were not statistically different from each other (difference = 0.052, SE = 0.061, *p* = .397), rejecting Hypothesis 1a.

Hypothesis 1b posited that mono‐minority identifiers prefer same‐ethnic dual identifiers over mono‐majority identifiers. The three most right estimates in Figure 1 show that mono‐minority identifiers showed no significant preference for mono‐majority identifiers but did so for same‐ethnic dual identifiers and same‐ethnic mono‐minority identifiers. The preference for same‐ethnic dual identifiers was significantly stronger than the preference for mono‐majority identifiers (difference = 0.454, SE = 0.162, *p* = .005), thus supporting Hypothesis 1b (see Online Supplement [Appendix 6 (Table A10)]).

Hypothesis 1c proposed that dual identifiers prefer mono‐majority identifiers, same‐ethnic dual identifiers, and same‐ethnic mono‐minority identifiers. Figure 1 reveals that dual identifiers only show a significant preference for same‐ethnic dual identifiers and same‐ethnic mono‐minority identifiers, as the line indicating dual identifiers' preference for mono‐majority identifiers is not above the baseline. Therefore, Hypothesis 1c is rejected.

In conclusion, dual identifiers did not show a higher likelihood of forming ties to multiple ethnic groups. While maintaining a preference for ties with same‐ethnic mono‐minority and (other) dual identifiers, they did not show such a preference for connections with the majority group. Neither did dual identifiers tend to befriend mono‐majority identifiers, nor did these mono‐majority identifiers exhibit a particularly strong preference for dual identifiers. However, our theoretical framework suggests that the potential of forming ties to multiple ethnic groups of dual identifiers varies depending on how their dual identities are cognitively constructed. Among the four types of dual identifiers, only compartmentalization dual identifiers are theorized to be more likely to form ties to multiple ethnic groups. To examine these group differences, SAOMs in which separate preferences for the different types of dual identifier were conducted next.

### Friendship preferences of different types of dual identifiers

**FIGURE 2:**
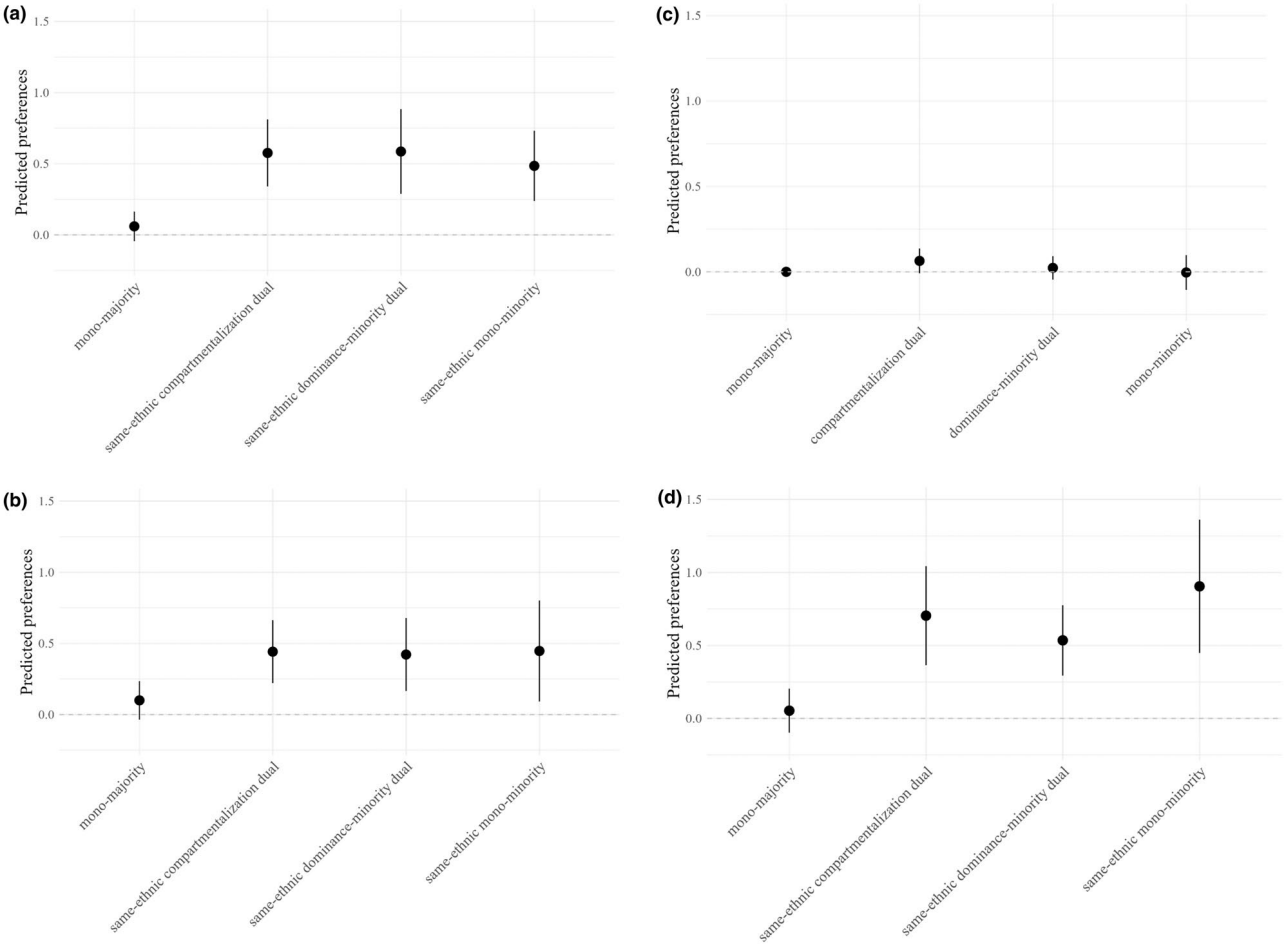
(a) Dominance‐minority dual identifiers' friendship preferences. (b) Compartmentalization dual identifiers' friendship preferences. (c) Mono‐majority identifiers' friendship preferences. (d) Mono‐minority dual identifiers' friendship preferences. (a–d) The friendship preferences are represented by point estimates along with 95% confidence intervals from meta‐analysis of 13 networks. The zero line denotes the reference category, friendships between mono‐majority identifiers. Differences between grade‐level networks and standard errors of estimates are reflected by the confidence intervals.

The full SAOM output is given in Online Supplement [Appendix 3 (Table A3)], which exhibited structural patterns consistent with those found in the previous analysis. To test Hypotheses 2, 3, 4, 5, group preferences were calculated as linear combination effects, as in the previous analysis (see Online Supplement [Appendix 5 (Table A8)]), and visualized in Figure 2a–d. See Online Supplement [Appendix 7 (Table A11)] for more information in the online supplement.

Hypothesis 2 anticipated that dominance‐minority dual identifiers would prefer students of the same minority ethnicity, including compartmentalization dual identifiers, other dominance‐minority identifiers, and mono‐minority identifiers. To test this hypothesis, we calculated dominance‐minority dual identifiers' preferences for all four groups and compared their preferences for the three groups to their preferences for mono‐majority identifiers. All three comparisons are significantly positive (see Online Supplement [Appendix 7 (Table A12)]); dominance‐minority dual identifier's preference for same‐ethnic compartmentalization dual identifiers versus dominance‐minority dual identifier's preference for mono‐majority identifiers: difference = 0.526, SE = 0. 101, *p* = .0001; dominance‐minority dual identifier's preference for same‐ethnic dominance‐minority dual identifiers versus dominance‐minority dual identifier's preference for mono‐majority identifiers: difference = 0.516, SE = 0.131, *p* = .001; dominance‐minority dual identifier's preference for same‐ethnic mono‐minority identifiers versus dominance‐minority dual identifier's preference for mono‐majority identifiers: difference = 0.425, SE = 0.137, *p* = .002, supporting Hypothesis 2. These results demonstrate that dominance‐minority dual identifiers had a stronger preference for students sharing the same minority ethnicity, regardless of whether they were mono‐minority identifiers or dual identifiers.

Hypothesis 3 posited that compartmentalization dual identifiers would prefer mono‐majority identifiers, same‐ethnic dual identifiers, and mono‐minority identifiers of the same ethnicity. However, as shown in Figure 2b, compartmentalization dual identifiers did not exhibit a significantly positive preference for mono‐majority identifiers. Instead, and in line with dominance‐minority dual identifiers, compartmentalization dual identifiers preferred friends from the same ethnicity, regardless of whether they were mono‐minority identifiers or dual identifiers. Accordingly, compartmentalization dual identifiers did not build connections between the two groups they self‐identified with. Therefore, Hypothesis 3 was rejected.

Hypothesis 4 predicted that mono‐majority identifiers would prefer mono‐majority identifiers, dominance‐majority dual identifiers, and compartmentalization dual identifiers. Given the exclusion of dominance‐majority dual identifiers, we could only test preferences for the other two groups. Mono‐majority identifiers' preference for their own group served as the baseline of the model. We found a trend toward a positive preference for compartmentalization dual identifiers on the *p* < .1 level (predicted preference = .064, SE = .037, *p* = .087; see Appendix 3 [Table A3]). However, the results did not show significant negative preferences for dominance‐minority or mono‐minority groups (see Figure 2c), indicating that the mono‐majority identifiers did not prefer mono‐majority identifiers and compartmentalization dual identifiers more than dominance‐minority and mono‐minority identifiers. This led to the rejection of Hypothesis 4.

Hypothesis 5 proposed that mono‐minority identifiers would prefer students who identify with the same ethnicity, including mono‐minority identifiers, dominance‐minority identifiers, and compartmentalization dual identifiers. Figure 2d shows that mono‐minority identifiers' preferences for same‐ethnic mono‐minority identifiers, dominance‐minority identifiers, and compartmentalization dual identifiers are significantly positive, while their preference for the mono‐majority group is non‐significant, supporting Hypothesis 5.

## CONCLUSION AND DISCUSSION

This study explored whether and how constructions of dual identity influenced dual identifiers' role in shaping interethnic friendship networks. Two SAOMs were employed modeling students' friendship selection longitudinally. The first model treated dual identifiers as a single group, while the second categorized them into four distinct types based on their conceptualization of their dual identity. Both models yielded consistent results: dual identifiers exhibited stronger preferences for other dual identifiers and mono‐minority identifiers of the same ethnicity rather than for mono‐majority identifiers. Meanwhile, mono‐majority identifiers did not clearly distinguish between dual and mono‐minority identifiers in their friendship selection; they preferred these groups just as much as other mono‐majority identifiers. Mono‐minority students, in contrast, showed stronger preferences for friendships with same‐ethnic dual identifiers than with mono‐majority identifiers.

These findings diverge from those reported by Jugert et al. (2018), who analyzed the same data, in two key ways. First, Jugert et al. (2018) concluded that dual identifiers tend to rather befriend mono‐majority identifying students than mono‐minority identifiers. Second, these authors found that mono‐minority identifiers preferred majority members as friends over other mono‐minority or dual identifying students. Both of these findings were contrary to Jugert et al.'s (2018) expectations, and we suspect that they are due to the fact that their analysis did not account for ethnic homophily in students' friendship decisions. While we modeled dual and mono‐minority identifiers choices to befriend peers of the *same* ethnicity, Jugert et al. (2018) modeled choices to befriend peers of *any* ethnic minority group. Incorporating ethnic homophily altered the interpretation of intergroup preferences and revealed that what may appear as a lack of preference in their study could manifest differently once ethnic homophily was formally considered in the model. In addition, thanks to advances in SAOM analyses, we were able to analyze 13 school‐grade networks, compared to Jugert et al.'s (2018) 9 networks. Given the considerable ethnic heterogeneity of the FIS data, analyses of more networks likely contributed to the divergent outcomes.

Importantly, the SAOM revealed that compartmentalization dual identifiers, dominance‐minority dual identifiers, and mono‐minority identifiers of the same ethnicity displayed strong preferences for friendships with each other, effectively excluding the mono‐majority group. This is in line with results of Leszczensky and Pink (2019), who found with the same dataset that students with strong ethnic identification preferred friends of the same ethnicity who were also strong ethnic identifiers. Despite having different attachments to the national identity, compartmentalization dual identifiers, dominance‐minority dual identifiers, and mono‐minority identifiers in our study showed similarly high levels of ethnic identification (see Table 4), making them equally attractive as friends. This suggests that dual identifiers' conceptualization of their dual identity did not lead to different behavioral tendencies in friendship formation.

Further, that mono‐minority identifiers did not differentiate in their friendship preferences between different types of dual identifiers implies that a stronger national identification is not penalized. Such a “black sheep effect” (Marques et al., 1988) has been observed in other contexts, where mono‐minority identifiers socially excluded those who were perceived as minority members but who self‐identified as belonging to the majority (Boda & Néray, 2015). As social identity theory (Tajfel & Turner, 1979) argues that people are motivated to maintain a positive social identity, an ingroup member who rejects the ingroup identity poses a threat to the group's image, leading to harsh evaluations of that deviating ingroup member. However, in our study, even those dual identifiers with the strongest national identification (compartmentalization) also exhibited high levels of ethnic identification, suggesting that they did not reject the ethnic ingroup. Accordingly, dual identifiers and same‐ethnic mono‐minority students exhibit strong mutual preference regardless of national identification strength, indicating that dual identifiers were not penalized for their host‐country identification.

In line with previous findings (Jugert et al., 2018; Leszczensky, 2013; Leszczensky et al., 2016) there was a tendency of majority group identifiers to prefer minority students with stronger national identification. In particular, we found that a marginally significant effect indicating that mono‐majority identifiers preferred compartmentalization dual identifiers, a type with relatively stronger national identification, as friends, but they were not more likely to befriend dominance‐minority dual identifiers or mono‐minority identifiers.

Importantly, the preference of majority group identifiers for dual identifiers with strong national identification was not reciprocated. Compartmentalization of dual identifiers did not capitalize on their theorized potential to form connections to the majority group. This finding fits work by Leszczensky (2013) and Leszczensky et al. (2016), who demonstrated that students with immigrant backgrounds and strong national identification did not exhibit a significantly stronger preference for natives than those with weaker national identification. This result might explain why dual identifiers were less likely to form ties to the two groups they belong to. Dual identifiers, no matter how they combined their different identities, only showed preferences for their same‐ethnic peers.

### Implications

These findings have several implications. Adolescents' cognitive organization of dual identity did not significantly correlate with their social behavior. Although different conceptualizations of dual identity (e.g., compartmentalization or dominance‐minority) were expected to shape social interactions, our results showed no substantial behavioral distinctions among these subgroups. Moreover, the expectation that dual identifiers facilitate minority–majority integration is challenged by our findings. Contrary to previous theoretical expectations (e.g., Levy et al., 2023; Love & Levy, 2019), dual identifiers did not reciprocate the majority group's preference and did not proactively form interethnic ties.

From a developmental perspective, these findings underscore the complexity of identity navigation during adolescence, a period marked by heightened identity exploration and increased reliance on peer relationships (Phinney, 1989, 2013; Umaña‐Taylor et al., 2014). Rather than serving as neutral or inclusive social brokers, dual identifiers may be engaging in selective identity‐based affiliation that reflects ongoing developmental negotiations around belonging and group membership (French et al., 2006). Their preference for same‐ethnic or other‐minority peers may provide social validation and reinforce a sense of shared experience in navigating marginalization or hybridity, especially in school contexts where ethnic hierarchies or exclusionary norms persist.

These network patterns align with recent evidence that adolescents' friendship networks are not only reflective of identity processes but also shape them over time (Kornienko et al., 2024; Rivas‐Drake et al., 2017). For dual identifiers, forming ties with other‐minority youth may support the development of an integrated or secure ethnic/national identity through shared social contexts and mutual recognition (Jugert et al., 2020). However, the relative absence of ties sent to mono‐majority identifiers raises questions about structural and relational constraints that may inhibit full inclusion, even among youth with dual affiliations. In this way, our study contributes to a growing understanding of how national and ethnic identity development unfolds not only within individuals but also through their embeddedness in dynamic peer networks.

### Limitations and future research

This study has several limitations that also suggest avenues for future research. First, while the dataset includes a substantial number of dual identifiers overall, the number of intersection and dominance‐majority dual identifiers per grade was often extremely small, limiting our ability to rigorously test hypotheses about these subgroups. Relatedly, although the sample included many dual identifiers, the relatively small proportion of mono‐minority students and their uneven distribution across ethnic groups created estimation challenges, including non‐convergence in some school‐grade networks. These constraints hindered our ability to detect nuanced effects involving minority actors. Future studies should seek larger and more balanced samples that allow for more fine‐grained comparisons between all identity groups.

Second, because minority students were intentionally oversampled, the mono‐majority group was not numerically dominant in many grades. This sampling strategy, while necessary to analyze dual identifiers, reduces the generalizability of our findings to school contexts where the majority group is demographically dominant. Future research should replicate this study in more representative school populations, both in Germany and across other European contexts, to assess the robustness and generalizability of the observed patterns.

Third, our study examines how self‐identified ethnicity shapes friendship choices, but does not address whether peers actually recognize these identities. While this assumption may hold for mono‐majority and mono‐minority students, it is less certain for dual identifiers whose identities may be less visible (Schlette et al., 2025). Future research should investigate how peer perceptions of ethnic identity intersect with self‐identification to influence network dynamics, for instance, whether mono‐majority students distinguish dual identifiers from mono‐minority peers and how such perceptions guide tie formation (see Boda, 2019; Boda et al., 2020; Boda & Néray, 2015).

Fourth, we treated ethnic self‐identification as static, even though adolescent identity development is dynamic and shaped by peer interactions (Branje et al., 2021). Although SAOM allows for modeling co‐evolution of networks and identities, such models are highly complex and beyond the scope of the current study. Nonetheless, future research should explore identity as an evolving construct within social networks, capturing the reciprocal influence between identity formation and peer relationships.

Fifth, while we accounted for factors like gender and school class as key drivers of adolescent homophily, we could not include other relevant individual‐level variables such as religion, socioeconomic status, or intergroup attitudes (e.g. Bergamaschi & Santagati, 2019; Block, 2015; Chabot, 2024; Cheadle & Schwadel, 2012; Killen et al., 2022; Kretschmer et al., 2024; MacInnis & Page‐Gould, 2015; Malacarne, 2017) due to model complexity and convergence issues. Although our focus was specifically on ethnic identification, we acknowledge that unobserved dimensions of similarity may also shape friendship choices. Future studies should integrate these factors to examine how various identity dimensions interact in structuring adolescent networks.

Sixth, due to small cell sizes and high heterogeneity, we were unable to include actor‐level covariates for specific ethnic groups (e.g., Turkish origin) or generational status (e.g., first‐ vs. second‐generation). Including such variables in our SAOMs led to convergence issues and overfitting risks. We therefore caution that our findings reflect broad patterns across diverse minority backgrounds, rather than effects specific to any one ethnic group. Future studies with larger and more homogeneous samples should investigate how dual identification functions within particular ethnic or migration‐origin groups.

Finally, while our study emphasized psychological and network mechanisms, identity and friendship choices are deeply embedded in structural and institutional contexts. In the German context, a substantial body of research has demonstrated how early tracking in education disproportionately disadvantages students with migration backgrounds (Krause & Schüller, 2014; Lüdemann & Schwerdt, 2013), while ethnic segregation in both neighborhoods and schools further limits opportunities for interethnic contact (Glitz, 2014; Kristen, 2008; Kruse, 2019). Additionally, structural racism and racialized school norms shape how minority and dual identity youth experience schooling and peer interactions (Hamed et al., 2020; Moffitt et al., 2019). These contextual forces influence not only who is available as a potential friend but also how youth interpret, express, and act upon their ethnic identities. Although we control for some contextual features, a more comprehensive account of peer relations should integrate developmental, structural, and network‐based perspectives. We encourage future research to bridge these levels of analysis in order to better understand how individual agency operates within broader systems of inequality.

### Conclusion

In conclusion, our findings challenge the assumption that dual identifiers are more likely to form interethnic connections to both the majority group and their ethnic minority group. Knowledge of dual identifiers may reduce prejudice among majority group members (Levy et al., 2023; Levy, Saguy, et al., 2017; Levy, Van Zomeren, et al., 2017) and, as we found, may even encourage majority group identifiers to befriend dual identifiers who strongly identify with the majority group. However, dual identifiers do not reciprocate such ties to the majority group. Instead, all types of dual identifiers tend to form connections with members of their own ethnicity or other ethnic minority groups, limiting their potential to facilitate minority‐majority integration.

## AUTHOR CONTRIBUTIONS

**Lexin Chen**: Conceptualization (equal); formal analysis (lead); methodology (lead); writing—original draft preparation (lead). **Tobias H. Stark**: Conceptualization (equal); formal analysis (supporting); methodology (supporting); write—review and editing (lead); supervision (equal); funding acquisition (lead). **Tom Nijs**: Conceptualization (equal); formal analysis (supporting); methodology (supporting); write—review and editing (equal); supervision (equal); project administration (lead). **Eva Jaspers**: Conceptualization (equal); formal analysis (supporting); methodology (supporting); write—review and editing (supporting); supervision (equal).

## FUNDING INFORMATION

This work was funded by the European Union (ERC, DUALNETS, 101043732). Views and opinions expressed are, however, those of the author(s) only and do not necessarily reflect those of the European Union or the European Research Council Executive Agency. Neither the European Union nor the granting authority can be held responsible for them.

## CONFLICT OF INTEREST STATEMENT

The authors declare no conflict of interest.

## ETHICS STATEMENT

The study was approved by the Ethics Committee of the Faculty of Social and Behavioral Sciences of Utrecht University (FETC 24‐0467).

## PATIENT CONSENT STATEMENT

Patient consent is not applicable. In order to carry out this study, no consent is needed.

## RIGHTS RETENTION STATEMENT

This work was funded by the European Union (ERC, DUALNETS, 101043732). As set out in the Grant Agreement, beneficiaries must ensure that at the latest at the time of publication, open access is provided via a trusted repository to the published version or the final peer‐reviewed manuscript accepted for publication under the latest available version of the Creative Commons Attribution International Public License (CC BY) or a license with equivalent rights. CC BY‐NC, CC BY‐ND, CC BY‐NC‐ND or equivalent licenses could be applied to long‐text formats.

## Supporting information


Data S1.

## Data Availability

The data that support the findings of this study are available from DeZIM‐Institut. Restrictions apply to the availability of these data, which were used under license for this study. Data are available from https://doi.org/10.34882/dezim.fis.c.1.0.0 with the permission of DeZIM‐Institut.
