# Supplementary material for: How does ethnic minority youth's dual self‐identification affect the formation of interethnic ties in friendship networks?
Source: J Res Adolesc. 2026 Mar 11;36(1):e70168. doi: 10.1111/jora.70168 (PMC12979707; doi:10.1111/jora.70168)
Supplement: Supplementary file 1 — Data S1. [file JORA-36-0-s001.docx]

**Online Supplement**

**Appendix 1: Measures for national and ethnic self-identification**

The independent variable in this study was self-identification, operationalized through responses to survey questions measuring both national and ethnic identification. Each construct was assessed using the mean score of six items adapted from Leszczensk & Santiago, (2015).

For national identification, adolescents responded the extent to which they agreed with the following statements: (1) “I am satisfied with being part of Germany”; (2) “I am happy to belong to Germany”; (3) “It bothers me when someone speaks negatively about Germany”; (4) “Germany is close to my heart”; (5) “I feel closely connected to Germans”; and (6) “I feel part of Germany.”

Similarly, ethnic identification was measured using parallel items reflecting the participant’s family’s country of origin: (1) “I am satisfied with being part of my family’s country of origin”; (2) “I am happy to belong to my family’s country of origin”; (3) “It bothers me when someone speaks negatively about my family’s country of origin”; (4) “My family’s country of origin is close to my heart”; (5) “I feel closely connected to people from my family’s country of origin”; and (6) “I feel part of my family’s country of origin.”

Responses for all items were recorded on a five-point Likert scale, ranging from 1 (“completely agree”) to 5 (“not applicable at all”). Responses were averaged with higher values indicating stronger national or ethnic identification.

**Appendix 2: Robustness tests results**

**Table A1**

*Meta-analysis SAOM results: Cancel the condition that the difference between national and ethnic identification strengths has to be less than 1*

| Effects | Estimates | Standard Error |
| --- | --- | --- |
| outdegree (density).(Intercept) | **-2.677***** | 0.079 |
| reciprocity.(Intercept) | **1.7***** | 0.088 |
| GWESP I -> K -> J (69).(Intercept) | **1.785***** | 0.046 |
| indegree - popularity.(Intercept) | **-0.068***** | 0.006 |
| outdegree - activity.(Intercept) | **0.017***** | 0.004 |
| indegree - activity.(Intercept) | **-0.138***** | 0.011 |
| m_minor alter.(Intercept) | -0.027 | 0.067 |
| m_minor ego.(Intercept) | 0.071 | 0.091 |
| d_minor alter.(Intercept) | 0.054 | 0.050 |
| d_minor ego.(Intercept) | 0.098 | 0.062 |
| d_comp alter.(Intercept) | 0.065 | 0.072 |
| d_comp ego.(Intercept) | 0.105 | 0.076 |
| gender alter.(Intercept) | -0.03 | 0.027 |
| gender ego.(Intercept) | 0.005 | 0.031 |
| same gender.(Intercept) | **0.468***** | 0.035 |
| same class.(Intercept) | **0.521***** | 0.027 |
| same ethnicity.(Intercept) | **0.169***** | 0.038 |
| reciprocity x GWESP I -> K -> J (69).(Intercept) | **-0.721***** | 0.075 |
| m_minor ego x m_minor alter.(Intercept) | 0.087 | 0.226 |
| m_minor ego x d_comp alter.(Intercept) | -0.123 | 0.265 |
| m_minor ego x same ethnicity.(Intercept) | 0.119 | 0.133 |
| m_minor alter x same ethnicity.(Intercept) | 0.148 | 0.143 |
| m_minor ego x d_minor alter.(Intercept) | -0.051 | 0.189 |
| d_minor alter x same ethnicity.(Intercept) | 0.11 | 0.101 |
| d_minor ego x d_comp alter.(Intercept) | -0.154 | 0.180 |
| d_minor ego x same ethnicity.(Intercept) | -0.095 | 0.084 |
| d_comp alter x same ethnicity.(Intercept) | -0.156 | 0.168 |
| d_minor ego x d_minor alter.(Intercept) | 0.248 | 0.143 |
| d_minor ego x m_minor alter.(Intercept) | 0.231 | 0.191 |
| d_comp ego x d_comp alter.(Intercept) | -0.246 | 0.208 |
| d_comp ego x same ethnicity.(Intercept) | 0.033 | 0.160 |
| d_comp ego x d_minor alter.(Intercept) | -0.092 | 0.153 |
| d_comp ego x m_minor alter.(Intercept) | -0.157 | 0.253 |
| d_minor ego x d_comp alter x same ethnicity.(Intercept) | **0.817*** | 0.369 |
| d_minor ego x d_minor alter x same ethnicity.(Intercept) | 0.265 | 0.225 |
| d_minor ego x m_minor alter x same ethnicity.(Intercept) | 0.229 | 0.337 |
| d_comp ego x d_comp alter x same ethnicity.(Intercept) | **1.093*** | 0.509 |
| d_comp ego x d_minor alter x same ethnicity.(Intercept) | 0.423 | 0.291 |
| d_comp ego x m_minor alter x same ethnicity.(Intercept) | 0.824 | 0.462 |
| m_minor ego x m_minor alter x same ethnicity.(Intercept) | 0.689 | 0.418 |
| m_minor ego x d_minor alter x same ethnicity.(Intercept) | 0.5 | 0.368 |
| m_minor ego x d_comp alter x same ethnicity.(Intercept) | **0.972*** | 0.457 |
| Note: *m_major*, mono-majority identifiers; *m_minor*, mono-minority identifiers; *d_comp*, compartmentalization dual identifiers; *d_minor*, dominance-minority dual identifiers; *GWESP*, geometrically weighted edgewise shared partners; † p < .1; * p < .05; ** p < .01; *** p < .001 | | |

**Table A2**

*Meta-analysis SAOM results: Lowering the cutoff point from 2 to 1.8 to distinguish weak and moderately weak identification*

| Effects | Estimates | Standard Error |
| --- | --- | --- |
| outdegree (density).(Intercept) | **-2.756***** | 0.114 |
| reciprocity.(Intercept) | **1.672***** | 0.077 |
| GWESP I -> K -> J (69).(Intercept) | **1.741***** | 0.054 |
| indegree - popularity.(Intercept) | **-0.059***** | 0.006 |
| outdegree - activity.(Intercept) | **0.019***** | 0.004 |
| indegree - activity.(Intercept) | **-0.135***** | 0.014 |
| m_minor alter.(Intercept) | 0.075 | 0.067 |
| m_minor ego.(Intercept) | 0.118 | 0.110 |
| d_minor alter.(Intercept) | 0.085 | 0.073 |
| d_minor ego.(Intercept) | 0.182 | 0.110 |
| d_comp alter.(Intercept) | **0.157**** | 0.058 |
| d_comp ego.(Intercept) | 0.18 | 0.107 |
| gender alter.(Intercept) | -0.019 | 0.038 |
| gender ego.(Intercept) | 0.001 | 0.037 |
| same gender.(Intercept) | **0.506***** | 0.037 |
| same class.(Intercept) | **0.513***** | 0.033 |
| same ethnicity.(Intercept) | **0.172***** | 0.043 |
| reciprocity x GWESP I -> K -> J (69).(Intercept) | **-0.717***** | 0.078 |
| m_minor ego x m_minor alter.(Intercept) | 0.074 | 0.319 |
| m_minor ego x d_comp alter.(Intercept) | -0.272 | 0.344 |
| m_minor ego x same ethnicity.(Intercept) | 0.13 | 0.212 |
| m_minor alter x same ethnicity.(Intercept) | -0.103 | 0.173 |
| m_minor ego x d_minor alter.(Intercept) | -0.149 | 0.278 |
| d_minor alter x same ethnicity.(Intercept) | 0.099 | 0.206 |
| d_minor ego x d_comp alter.(Intercept) | -0.033 | 0.299 |
| d_minor ego x same ethnicity.(Intercept) | -0.107 | 0.091 |
| d_comp alter x same ethnicity.(Intercept) | **-0.352*** | 0.164 |
| d_minor ego x d_minor alter.(Intercept) | -0.017 | 0.267 |
| d_minor ego x m_minor alter.(Intercept) | 0.05 | 0.285 |
| d_comp ego x d_comp alter.(Intercept) | -0.186 | 0.210 |
| d_comp ego x same ethnicity.(Intercept) | -0.142 | 0.176 |
| d_comp ego x d_minor alter.(Intercept) | -0.03 | 0.205 |
| d_comp ego x m_minor alter.(Intercept) | 0.017 | 0.263 |
| d_minor ego x d_comp alter x same ethnicity.(Intercept) | 0.585 | 0.376 |
| d_minor ego x d_minor alter x same ethnicity.(Intercept) | 0.607 | 0.490 |
| d_minor ego x m_minor alter x same ethnicity.(Intercept) | 0.477 | 0.477 |
| d_comp ego x d_comp alter x same ethnicity.(Intercept) | 0.606 | 0.455 |
| d_comp ego x d_minor alter x same ethnicity.(Intercept) | 0.368 | 0.504 |
| d_comp ego x m_minor alter x same ethnicity.(Intercept) | 0.134 | 0.495 |
| m_minor ego x m_minor alter x same ethnicity.(Intercept) | 0.18 | 0.523 |
| m_minor ego x d_minor alter x same ethnicity.(Intercept) | 0.415 | 0.494 |
| m_minor ego x d_comp alter x same ethnicity.(Intercept) | 0.838 | 0.511 |
| Note: *m_major*, mono-majority identifiers; *m_minor*, mono-minority identifiers; *d_comp*, compartmentalization dual identifiers; *d_minor*, dominance-minority dual identifiers; *GWESP*, geometrically weighted edgewise shared partners; † p < .1; * p < .05; ** p < .01; *** p < .001 | | |

**Appendix 3: SAOM Result: Classifying dual identifiers**

**Table A3**

*Meta-analysis SAOM results: Classifying dual identifiers*

| Effects | Estimates | Standard Error |
| --- | --- | --- |
| outdegree (density) | **-2.646***** | 0.074 |
| reciprocity | **1.665***** | 0.075 |
| GWESP I -> K -> J (69) | **1.775***** | 0.046 |
| reciprocity x GWESP I -> K -> J (69) | **-0.700***** | 0.064 |
| indegree - popularity | **-0.068***** | 0.006 |
| outdegree - activity | **0.015***** | 0.003 |
| indegree - activity | **-0.135***** | 0.010 |
| m_minor alter | -0.004 | 0.052 |
| m_minor ego | 0.053 | 0.077 |
| d_minor alter | 0.023 | 0.035 |
| d_minor ego | 0.060 | 0.053 |
| d_comp alter | **0.064†** | 0.037 |
| d_comp ego | 0.100 | 0.069 |
| gender alter | 0.010 | 0.011 |
| gender ego | **-0.023*** | 0.011 |
| same gender | **0.463***** | 0.032 |
| same class | **0.522***** | 0.026 |
| same ethnicity | **0.170***** | 0.037 |
| m_minor ego x m_minor alter | 0.072 | 0.160 |
| m_minor ego x d_comp alter | -0.129 | 0.144 |
| m_minor ego x same ethnicity | 0.101 | 0.104 |
| m_minor alter x same ethnicity | 0.031 | 0.102 |
| m_minor ego x d_minor alter | 0.001 | 0.125 |
| d_minor alter x same ethnicity | 0.125 | 0.088 |
| d_minor ego x d_comp alter | 0.039 | 0.103 |
| d_minor ego x same ethnicity | -0.053 | 0.047 |
| d_comp alter x same ethnicity | -0.113 | 0.071 |
| d_minor ego x d_minor alter | 0.106 | 0.086 |
| d_minor ego x m_minor alter | 0.088 | 0.128 |
| d_comp ego x d_comp alter | -0.093 | 0.083 |
| d_comp ego x same ethnicity | -0.092 | 0.080 |
| d_comp ego x d_minor alter | 0.021 | 0.077 |
| d_comp ego x m_minor alter | 0.028 | 0.121 |
| d_minor ego x d_comp alter x same ethnicity | **0.377**** | 0.121 |
| d_minor ego x d_minor alter x same ethnicity | 0.099 | 0.169 |
| d_minor ego x m_minor alter x same ethnicity | 0.152 | 0.173 |
| d_comp ego x d_comp alter x same ethnicity | **0.271†** | 0.153 |
| d_comp ego x d_minor alter x same ethnicity | 0.062 | 0.120 |
| d_comp ego x m_minor alter x same ethnicity | 0.259 | 0.261 |
| m_minor ego x m_minor alter x same ethnicity | 0.354 | 0.323 |
| m_minor ego x d_minor alter x same ethnicity | 0.107 | 0.166 |
| m_minor ego x d_comp alter x same ethnicity | **0.688**** | 0.241 |
| Note: *m_major*, mono-majority identifiers; *m_minor*, mono-minority identifiers; *d_comp*, compartmentalization dual identifiers; *d_minor*, dominance-minority dual identifiers; *GWESP*, geometrically weighted edgewise shared partners; † p < .1; * p < .05; ** p < .01; *** p < .001 | | |

**Appendix 4: Grade-level descriptives of SAOM**

**Table A4**

*Grade-level descriptives: wave 1*

| Grade | 1 | 3 | 7 | 8 | 11 | 13 | 15 | 18 | 22 | 23 | 24 | 26 | 27 |
| --- | --- | --- | --- | --- | --- | --- | --- | --- | --- | --- | --- | --- | --- |
| Ethnic distribution |  |  |  |  |  |  |  |  |  |  |  |  |  |
| Germany | 17 | 13 | 11 | 20 | 34 | 52 | 27 | 55 | 20 | 37 | 40 | 27 | 8 |
| Turkey | 14 | 14 | 25 | 18 | 29 | 12 | 28 | 6 | 38 | 33 | 26 | 10 | 9 |
| Former Soviet Union | 4 | 4 | 3 | 7 | 5 | 0 | 0 | 18 | 9 | 8 | 5 | 2 | 0 |
| Poland | 3 | 4 | 5 | 3 | 3 | 3 | 2 | 2 | 2 | 8 | 10 | 5 | 0 |
| Italy | 2 | 4 | 1 | 2 | 1 | 7 | 1 | 0 | 2 | 3 | 0 | 1 | 0 |
| Number of students | 54 | 50 | 63 | 78 | 88 | 82 | 73 | 100 | 81 | 92 | 87 | 49 | 29 |
| Number of friends | 389 | 309 | 507 | 526 | 620 | 604 | 587 | 639 | 559 | 577 | 513 | 335 | 299 |
| Number of ties from mono-majority to mono-majority | 23 | 19 | 7 | 24 | 79 | 169 | 39 | 184 | 18 | 110 | 110 | 85 | 15 |
| Number of ties from mono-majority to dual | 43 | 29 | 43 | 66 | 73 | 63 | 49 | 88 | 44 | 100 | 62 | 49 | 13 |
| Number of ties from mono-majority to mono-minority | 17 | 10 | 6 | 4 | 6 | 6 | 6 | 10 | 2 | 9 | 1 | 8 | 2 |
| Number of ties from dual to mono-majority | 44 | 39 | 39 | 43 | 69 | 70 | 67 | 96 | 54 | 87 | 77 | 51 | 14 |
| Number of ties from dual to dual | 112 | 65 | 151 | 193 | 124 | 37 | 111 | 62 | 175 | 115 | 100 | 38 | 36 |
| Number of ties from dual to mono-minority | 13 | 13 | 15 | 10 | 18 | 6 | 11 | 8 | 8 | 11 | 4 | 7 | 2 |
| Number of ties from mono-minority to mono-majority | 12 | 6 | 7 | 0 | 4 | 4 | 4 | 10 | 3 | 3 | 3 | 0 | 3 |
| Number of ties from mono-minority to dual | 22 | 6 | 15 | 15 | 33 | 5 | 8 | 3 | 10 | 14 | 8 | 0 | 4 |
| Number of ties from mono-minority to mono-minority | 5 | 2 | 4 | 0 | 4 | 1 | 0 | 0 | 0 | 0 | 1 | 0 | 0 |

**Table A5**

*Grade-level descriptives: wave 2*

| Grade | 1 | 3 | 7 | 8 | 11 | 13 | 15 | 18 | 22 | 23 | 24 | 26 | 27 |
| --- | --- | --- | --- | --- | --- | --- | --- | --- | --- | --- | --- | --- | --- |
| Ethnic distribution |  |  |  |  |  |  |  |  |  |  |  |  |  |
| Germany | 11 | 12 | 9 | 19 | 44 | 54 | 29 | 54 | 24 | 39 | 36 | 24 | 9 |
| Turkey | 15 | 12 | 26 | 16 | 31 | 12 | 32 | 6 | 47 | 34 | 33 | 9 | 10 |
| Former Soviet Union | 6 | 4 | 3 | 7 | 4 | 1 | 0 | 20 | 11 | 8 | 5 | 2 | 0 |
| Poland | 4 | 3 | 6 | 5 | 4 | 3 | 3 | 5 | 4 | 8 | 9 | 3 | 5 |
| Italy | 3 | 2 | 5 | 3 | 1 | 7 | 3 | 0 | 4 | 3 | 3 | 1 | 0 |
| Number of students | 53 | 49 | 68 | 77 | 100 | 95 | 93 | 108 | 98 | 98 | 92 | 44 | 48 |
| Number of friends | 357 | 274 | 477 | 479 | 650 | 574 | 601 | 672 | 556 | 642 | 534 | 277 | 258 |
| Number of ties from mono-majority to mono-majority | 12 | 9 | 8 | 31 | 160 | 173 | 52 | 197 | 46 | 96 | 101 | 70 | 16 |
| Number of ties from mono-majority to dual | 36 | 13 | 22 | 50 | 78 | 75 | 71 | 120 | 80 | 86 | 56 | 30 | 26 |
| Number of ties from mono-majority to mono-minority | 11 | 1 | 9 | 6 | 13 | 17 | 10 | 15 | 6 | 12 | 12 | 3 | 6 |
| Number of ties from dual to mono-majority | 39 | 18 | 31 | 44 | 68 | 85 | 55 | 118 | 63 | 79 | 68 | 30 | 20 |
| Number of ties from dual to dual | 122 | 38 | 171 | 141 | 81 | 49 | 148 | 81 | 190 | 164 | 133 | 25 | 25 |
| Number of ties from dual to mono-minority | 21 | 28 | 47 | 21 | 30 | 15 | 26 | 4 | 22 | 15 | 26 | 5 | 12 |
| Number of ties from mono-minority to mono-majority | 16 | 4 | 11 | 8 | 14 | 17 | 6 | 15 | 7 | 13 | 3 | 1 | 9 |
| Number of ties from mono-minority to dual | 29 | 22 | 39 | 37 | 29 | 15 | 17 | 4 | 24 | 15 | 17 | 0 | 8 |
| Number of ties from mono-minority to mono-minority | 10 | 9 | 11 | 7 | 10 | 8 | 1 | 3 | 2 | 1 | 6 | 0 | 5 |

**Table A6**

*Grade-level descriptives: wave 3*

| Grade | 1 | 3 | 7 | 8 | 11 | 13 | 15 | 18 | 22 | 23 | 24 | 26 | 27 |
| --- | --- | --- | --- | --- | --- | --- | --- | --- | --- | --- | --- | --- | --- |
| Ethnic distribution |  |  |  |  |  |  |  |  |  |  |  |  |  |
| Germany | 13 | 8 | 14 | 22 | 41 | 49 | 27 | 48 | 30 | 36 | 39 | 19 | 11 |
| Turkey | 18 | 16 | 24 | 18 | 38 | 13 | 25 | 7 | 49 | 37 | 34 | 11 | 11 |
| Former Soviet Union | 6 | 5 | 1 | 5 | 5 | 0 | 1 | 20 | 12 | 6 | 5 | 2 | 1 |
| Poland | 3 | 3 | 6 | 2 | 6 | 2 | 3 | 6 | 2 | 8 | 9 | 4 | 3 |
| Italy | 3 | 3 | 2 | 2 | 2 | 7 | 3 | 1 | 1 | 1 | 3 | 0 | 0 |
| Number of students | 59 | 54 | 67 | 74 | 105 | 91 | 85 | 109 | 104 | 99 | 96 | 44 | 49 |
| Number of friends | 338 | 219 | 406 | 475 | 718 | 560 | 579 | 630 | 560 | 620 | 540 | 275 | 237 |
| Number of ties from mono-majority to mono-majority | 14 | 5 | 14 | 34 | 149 | 159 | 66 | 174 | 54 | 104 | 103 | 36 | 12 |
| Number of ties from mono-majority to dual | 36 | 13 | 23 | 34 | 82 | 84 | 52 | 73 | 77 | 69 | 55 | 27 | 21 |
| Number of ties from mono-majority to mono-minority | 19 | 2 | 8 | 4 | 33 | 3 | 4 | 13 | 6 | 10 | 2 | 3 | 2 |
| Number of ties from dual to mono-majority | 24 | 23 | 27 | 12 | 51 | 65 | 48 | 85 | 75 | 55 | 61 | 18 | 21 |
| Number of ties from dual to dual | 72 | 47 | 59 | 116 | 71 | 53 | 109 | 56 | 209 | 160 | 101 | 20 | 51 |
| Number of ties from dual to mono-minority | 45 | 11 | 28 | 28 | 55 | 11 | 11 | 10 | 12 | 30 | 19 | 0 | 4 |
| Number of ties from mono-minority to mono-majority | 18 | 1 | 10 | 2 | 17 | 6 | 3 | 13 | 4 | 9 | 2 | 0 | 3 |
| Number of ties from mono-minority to dual | 42 | 15 | 19 | 27 | 59 | 13 | 16 | 6 | 13 | 32 | 23 | 0 | 7 |
| Number of ties from mono-minority to mono-minority | 25 | 5 | 16 | 4 | 37 | 6 | 0 | 2 | 0 | 7 | 2 | 0 | 1 |

**Appendix 5: Calculating group preferences through linear combination effects**

To test Hypotheses 1(a, b, c) and 2-5, it is necessary to compare groupwise friendship preferences, accounting for the ethnic homophily effect. In the first SAOM, for example, the preference of dual identifiers for mono-minority identifiers of the same ethnicity is calculated by linearly combining seven components: the ego effect of dual identifiers, the alter effect of mono-minority identifiers, the same-ethnicity effect, the interaction between the dual-identifier ego effect and the same-ethnicity effect, the interaction between the mono-minority identifier alter effect and the same-ethnicity effect, the interaction between the dual-identifier ego effect and the mono-minority identifier alter effect, and the three-way interaction among these effects. These components are scaled and linearly combined before conducting the meta-analysis. See Table A7 for more details.

In the second SAOM, for instance, dominance-minority dual identifier group’s preference for same-ethnic mono-minority identifiers is calculated by linearly combining seven components: the ego effect of dominance-minority dual identifiers, the alter effect of mono-minority identifiers, the same-ethnicity effect, the interaction between the ego effect of dominance-minority dual identifiers and the same-ethnicity effect, the interaction between the mono-minority identifier alter effect and the same-ethnicity effect, the interaction between the ego effect of dominance-minority dual identifiers and the alter effect of mono-minority identifiers, and the three-way interaction among these effects. These components are scaled and linearly combined before conducting the meta-analysis. See Table A8 for more details.

**Table A7**

*Needed effects for linear combination to calculate group preferences: Dual Identifiers as A Whole Group*

| Preferences | Needed effects |
| --- | --- |
| **Dual identifiers** |  |
| dual -> mono-majority | ego(dual identifier) |
| dual -> dual of the same ethnicity | same(ethnicity) + ego(dual identifier) + alter(dual identifier) + ego(dual identifier) * same(ethnicity) + alter(dual identifier) * same(ethnicity)  + ego(dual identifier) * alter(dual identifier) * same(ethnicity) |
| dual -> mono-minority of the same ethnicity | same(ethnicity) + ego(dual identifier) + alter(mono-minority identifier) +  ego(dual identifier) * alter(mono-minority identifier) + ego(dual identifier)*same(ethnicity) +  alter(mono-minority identifier) * same(ethnicity) + ego(dual identifier) * alter(mono-minority identifier) * same(ethnicity) |
| **Mono-majority identifiers** |  |
| mono-majority -> mono-majority | **baseline** |
| mono-majority -> dual | alter(dual identifier) |
| mono-majority -> mono-minority | alter(mono-minority identifier) |
| **Mono-minority identifiers** |  |
| mono-minority -> mono-majority | ego(mono-minority identifier) |
| mono-minority -> dual | same(ethnicity) + ego(mono-minority identifier) + alter(dual identifier) + ego(mono-minority identifier) * alter(dual identifier) + ego(mono-minority identifier) * same(ethnicity) + alter(dual identifier)*same(ethnicity) + ego(mono-minority identifier) * alter(dual identifier) * same(ethnicity) |
| mono-minority -> mono-minority | same(ethnicity) + ego(mono-minority identifier) + alter(mono-minority identifier) + ego(mono-minority identifier) * alter(mono-minority identifier) + ego(mono-minority identifier) * same(ethnicity) + alter(mono-minority identifier)*same(ethnicity) + ego(mono-minority identifier) * alter(mono-minority identifier) * same(ethnicity) |
| Note: *dual*, dual identifiers | |

**Table A8**

*Needed effects for linear combination to calculate group preferences: Classifying dual Identifiers*

|  | Needed effects |
| --- | --- |
| **Compartmentalization Dual identifiers(d_comp)** |  |
| d_comp -> d_comp of the same ethnicity | same(ethnicity) + ego(d_comp identifier) + alter(d_comp identifier) + same(ethnicity) * ego(d_comp identifier) + same(ethnicity) * alter(d_comp identifier) + ego(d_comp identifier) * alter(d_comp identifier) + same(ethnicity) * ego(d_comp identifier) * alter(d_comp identifier) |
| d_comp -> d_minor of the same ethnicity | same(ethnicity) + ego(d_comp identifier) + alter(d_minor identifier) + same(ethnicity) * ego(d_comp identifier) + same(ethnicity) * alter(d_minor identifier) + ego(d_comp identifier) * alter(d_minor identifier) + same(ethnicity) * ego(d_comp identifier) * alter(d_minor identifier) |
| d_comp -> m_major | ego(d_comp identifier) |
| d_comp -> m_minor of the same ethnicity | same(ethnicity) + ego(d_comp identifier) + alter(m_minor identifier) + same(ethnicity) * ego(d_comp identifier) + same(ethnicity) * alter(m_minor identifier) + ego(d_comp identifier) * alter(m_minor identifier) + same(ethnicity) * ego(d_comp identifier) * alter(m_minor identifier) |
| **Dominance-minority Dual identifiers(d_minor)** |  |
| d_minor -> d_comp of the same ethnicity | same(ethnicity) + ego(d_minor identifier) + alter(d_comp identifier) + same(ethnicity) * ego(d_minor identifier) + same(ethnicity) * alter(d_comp identifier) + ego(d_minor identifier) * alter(d_comp identifier) + same(ethnicity) * ego(d_minor identifier) * alter(d_comp identifier) |
| d_minor -> d_minor of the same ethnicity | same(ethnicity) + ego(d_minor identifier) + alter(d_minor identifier) + same(ethnicity) * ego(d_minor identifier) + same(ethnicity) * alter(d_minor identifier) + ego(d_minor identifier) * alter(d_minor identifier) + same(ethnicity) * ego(d_minor identifier) * alter(d_minor identifier) |
| d_minor -> m_major | ego(d_minor identifier) |
| d_minor -> m_minor of the same ethnicity | same(ethnicity) + ego(d_minor identifier) + alter(m_minor identifier) + same(ethnicity) * ego(d_minor identifier) + same(ethnicity) * alter(m_minor identifier) + ego(d_minor identifier) * alter(m_minor identifier) + same(ethnicity) * ego(d_minor identifier) * alter(m_minor identifier) |
| **Mono-majority identifiers(m_major)** |  |
| m_major -> d_comp | alter(d_comp identifier) |
| m_major -> d_minor | alter(d_minor identifier) |
| m_major -> m_major | **baseline** |
| m_major -> m_minor | alter(m_minor identifier) |
| **Mono-minority identifiers(m_minor)** |  |
| m_minor -> d_comp of the same ethnicity | same(ethnicity) + ego(m_minor identifier) + alter(d_comp identifier) + same(ethnicity) * ego(m_minor identifier) + same(ethnicity) * alter(d_comp identifier) + ego(m_minor identifier) * alter(d_comp identifier) + same(ethnicity) * ego(m_minor identifier) * alter(d_comp identifier) |
| m_minor -> d_minor of the same ethnicity | same(ethnicity) + ego(m_minor identifier) + alter(d_minor identifier) + same(ethnicity) * ego(m_minor identifier) + same(ethnicity) * alter(d_minor identifier) + ego(m_minor identifier) * alter(d_minor identifier) + same(ethnicity) * ego(m_minor identifier) * alter(d_minor identifier) |
| m_minor -> m_major | ego(m_minor identifier) |
| m_minor -> m_minor of the same ethnicity | same(ethnicity) + ego(m_minor identifier) + alter(m_minor identifier) + same(ethnicity) * ego(m_minor identifier) + same(ethnicity) * alter(m_minor identifier) + ego(m_minor identifier) * alter(m_minor identifier) + same(ethnicity) * ego(m_minor identifier) * alter(m_minor identifier) |
| Note: *m_major*, mono-majority identifiers; *m_minor*, mono-minority identifiers; *d_comp*, compartmentalization dual identifiers; *d_minor*, dominance-minority dual identifiers | |

**Appendix 6: Calculating and comparing group preferences for the first SAOM**

**Table A9**

*Group Preferences (Linear Combination effects): Dual Identifiers as A Whole Group*

|  | Estimates | S.E. |
| --- | --- | --- |
| **Dual identifiers** |  |  |
| dual -> mono-majority | 0.062 | 0.038 |
| dual -> dual of the same ethnicity | **0.381***** | 0.085 |
| dual -> mono-minority of the same ethnicity | **0.424***** | 0.093 |
| **Mono-majority identifiers** |  |  |
| mono-majority -> mono-majority | baseline | |
| mono-majority -> dual | 0.018 | 0.020 |
| mono-majority -> mono-minority | -0.034 | 0.058 |
| **Mono-minority identifiers** |  |  |
| mono-minority -> mono-majority | 0.096 | 0.077 |
| mono-minority -> dual | **0.550***** | 0.143 |
| mono-minority -> mono-minority | **0.979***** | 0.224 |
| Note: *dual*, dual identifiers; † p < .1; * p < .05; ** p < .01; *** p < .001 | | |

**Table A10**

*Hypotheses 1(a, b) Z-test Comparison Results*

|  | Estimates | S.E. |
| --- | --- | --- |
| **Hypothesis 1a: Mono-majority identifiers** |  |  |
| m_major -> dual vs. m_major -> m_minor | 0.052 | 0.061 |
| **Hypothesis 1b: Mono-minority identifiers** |  |  |
| m_minor -> dual vs. m_minor -> m_major | **0.454**** | 0.162 |
| Note: *m_major*, mono-majority identifiers; *m_minor*, mono-minority identifiers; *dual*, dual identifiers; † p < .1; * p < .05; ** p < .01; *** p < .001 | | |

**Appendix 7: Calculating and comparing group preferences for the second SAOM**

**Table A11**

*Group preferences (Linear combination effects): Classifying dual identifiers*

| Group preference | Estimates | S.E. |
| --- | --- | --- |
| **Compartmentalization dual identifiers** |  |  |
| d_comp -> m_major | 0.100 | 0.069 |
| d_comp -> d_comp of the same ethnicity | **0.442***** | 0.113 |
| d_comp -> d_minor of the same ethnicity | **0.422**** | 0.131 |
| d_comp -> m_minor of the same ethnicity | **0.447*** | 0.181 |
| **Dominance-minority dual identifiers** |  |  |
| d_minor -> m_major | 0.060 | 0.053 |
| d_minor -> d_comp of the same ethnicity | **0.576***** | 0.120 |
| d_minor -> d_minor of the same ethnicity | **0.586***** | 0.152 |
| d_minor -> m_minor of the same ethnicity | **0.485**** | 0.126 |
| **Mono-majority identifiers** |  |  |
| m_major -> m_major | baseline | |
| m_major -> d_comp | **0.064†** | 0.037 |
| m_major -> d_minor | 0.023 | 0.035 |
| m_major -> m_minor | -0.004 | 0.052 |
| **Mono-minority identifiers** |  |  |
| m_minor -> m_major | 0.053 | 0.077 |
| m_minor -> d_comp of the same ethnicity | **0.704***** | 0.173 |
| m_minor -> d_minor of the same ethnicity | **0.535***** | 0.123 |
| m_minor -> m_minor of the same ethnicity | **0.905***** | 0.233 |
| Note: *m_major*, mono-majority identifiers; *m_minor*, mono-minority identifiers; *d_comp*, compartmentalization dual identifiers; *d_minor*, dominance-minority dual identifiers; † p < .1; * p < .05; ** p < .01; *** p < .001 | | |

**Table A12**

*Hypothesis 2 z-test comparison results*

| **Hypothesis 2: Dominance-minority dual identifiers** | Estimates | S.E. |
| --- | --- | --- |
| d_minor -> d_minor vs. d_minor -> m_major | **0.526**** | 0.101 |
| d_minor -> d_comp vs. d_minor -> m_major | **0.516***** | 0.131 |
| d_minor -> m_minor vs. d_minor -> m_major | **0.425**** | 0.137 |
| Note: *m_major*, mono-majority identifiers; *m_minor*, mono-minority identifiers; *d_comp*, compartmentalization dual identifiers; *d_minor*, dominance-minority dual identifiers; † p < .1; * p < .05; ** p < .01; *** p < .001 | | |

**Reference**

Leszczensk, L., & Santiago, A. G. (2015). The Developement and Test of a Measure of Youth’s Ethnic and National Identity. *Methods, Data, Analyses*, *9*(1), 24.

Wasserman, S. (1994). Social network analysis: Methods and applications. *The Press Syndicate of the University of Cambridge*.
